# ‘Metal Complexes as Ligands’ for the Synthesis of Coordination Polymers: A Mn^III^ Monomer as a Building Block for the Preparation of an Unprecedented 1-D {Mn^II^Mn^III^}_n_ Linear Chain

**DOI:** 10.3390/ma13061352

**Published:** 2020-03-17

**Authors:** Konstantinos N. Pantelis, Georgios Karotsis, Christos Lampropoulos, Luís Cunha-Silva, Albert Escuer, Theocharis C. Stamatatos

**Affiliations:** 1 Chemistry Department, University of Patras, 265 04 Patras, Greece; kostaspantelis95@gmail.com; 2 Department of Chemistry, 1812 Sir Isaac Brock Way, Brock University, L2S 3A1 St. Catharines, ON L2S 3A1, Canada; g.karotsis@utah.edu; 3 Department of Chemistry, University of North Florida, 1 UNF Dr., Jacksonville, FL 32224, USA; christosl1981@gmail.com; 4 LAQV/REQUIMTE & Department of Chemistry and Biochemistry, Faculty of Sciences, University of Porto, 4169-007 Porto, Portugal; l.cunha.silva@fc.up.pt; 5 Departament de Quimica Inorgànica i Orgànica, Secció Inorgànica and Institut de Nanociència i Nanotecnologia (IN2UB), Universitat de Barcelona, Martí Franqués 1-11, 08028 Barcelona, Spain; albert.escuer@qi.ub.es

**Keywords:** manganese, coordination polymers, single-crystal X-ray crystallography, Schiff bases, metalloligands, magnetic properties, supramolecular chemistry

## Abstract

A relatively unexplored synthetic route in redox-active Mn(II/III) coordination chemistry has been employed toward the preparation of a new mixed-valence Mn^II/III^ 1-D linear chain from the reaction of [Mn^III^(sacb)_2_]^−^ precursor with a Mn^II^ source, where sacbH_2_ is the Schiff base ligand *N*-salicylidene-2-amino-5-chlorobenzoic acid. The mononuclear (Pr_2_NH_2_)[Mn^III^(sacb)_2_] (**1**) compound was obtained in excellent yields (>85%) from the 1:2:3 reaction of Mn(O_2_CMe)_2_∙4H_2_O, sacbH_2_ and Pr_2_NH, respectively. In **1**, the two doubly deprotonated sacb^2−^ ligands act as O_carboxylate_,N_imine_,O_phenoxide_-tridentate chelates, while the second carboxylate O atom of sacb^2−^ is dangling and H-bonded to the Pr_2_NH_2_^+^ countercation. Complex **1** was subsequently used as a ‘ligand’ to react stoichiometrically with the ‘metal’ Mn(NO_3_)_2_∙4H_2_O, thus leading to the 1-D coordination polymer {[Mn^II^Mn^III^(sacb)_2_(H_2_O)_2_(MeOH)_2_](NO_3_)}_n_ (**2**) in good yields (~50%). The removal of Pr_2_NH_2_^+^ from the vicinity of the [Mn^III^(sacb)_2_]^−^ metalloligand has rendered possible (vide infra) the coordination of the second O_carboxylate_ of sacb^2−^ to neighboring {Mn^II^(H_2_O)_2_(MeOH)_2_}^2+^ units, and consequently the formation of the 1-D polymer **2**. Direct-current (dc) magnetic susceptibility studies revealed the presence of very weak antiferromagnetic exchange interactions between alternating Mn^III^ and Mn^II^ atoms with a coupling constant of *J* = −0.08 cm^−1^ for *g* = 2.00. The combined results demonstrate the potential of the ‘metal complexes as ligands’ approach to yield new mixed-valence Mn(II/III) coordination polymers with interesting structural motifs and physicochemical properties.

## 1. Introduction

The synthesis of new polynuclear complexes and coordination polymers of Mn in moderate to high oxidation states (i.e., II, III, and IV) continues to receive a great deal of attention as a route to molecule-based materials with interesting structures, and possible function as nanoscale magnetic particles or nanowires [1]. The former compounds belong to the class of single-molecule magnets (SMMs) [2], which are molecular species that have a significant barrier to magnetization relaxation arising from a combination of a large ground-state spin, *S*, and an eas*y*-axis anisotropy (negative zero-field splitting (ZFS) parameter, *D*) [3]. On the other hand, one-dimensional (1-D) coordination polymers of Mn^III^-containing atoms could behave as single-chain magnets (SCMs) [4], provided that they possess a large uniaxial anisotropy, strong intra-chain exchange interactions without spin compensation between the high-spin magnetic units, and good isolation of the chains in order to avoid two- and three-dimensional ordering [5]. These features can lead to an upper limit to the relaxation barrier (*Δ*) given by (*D*+4*J*)*S*^2^, where *J* is the interaction between repeating units of the chain [6]. Both SMMs and SCMs are of great interest because of their unusual physical properties, and their potential use in information storage at the molecular level and as qubits in quantum computation [7]. There are now many 3d-metal SMMs [8], but the number of SCMs is still relatively small [9], even though the first one was discovered many years ago [10]. Of the currently known examples, the majority are heterospin systems containing at least two different spin carriers bridged by organic radicals [10,11], oximate [12] or Prussian blue anions and derivatives [13], and obtained by a direct approach, using SMMs as building blocks. Homospin SCMs are rare, and most of them have been synthesized serendipitously by using highly anisotropic metal sources, such as Mn^III^ [14], Fe^II/III^ [15], and Co^II^ ions [16].

Given the scarcity of commercially available Mn^III^ starting materials, one of the challenges in synthetic inorganic chemistry is to find new methods or develop relatively unexplored strategies for the stabilization of Mn ions in the magnetically interesting—for SMM and SCM applications—3+ oxidation state [17]. Several methods have been successfully employed over the past 20 years or so, but the self-assembly approach still remains the most widely used synthetic route. This procedure utilizes a variety of potentially chelating and simultaneously bridging organic and/or inorganic ligands, which can foster the mild oxidation of Mn^II^ to Mn^III^, usually provoked by atmospheric O_2_ under ambient conditions, and provide the resulting molecular systems with thermodynamic stability and crystallinity [1]. Another method, albeit much less employed, for the isolation of polynuclear Mn^III^-containing complexes and coordination polymers is the ‘metal complexes as ligands’ strategy [18]. This route makes use of small in nuclearity Mn^III^ complexes (usually monomers, dimers, or trimers) with coordinately unsaturated ligands, which have donor atoms readily available for binding to additional metal ions.

**Scheme 1:**
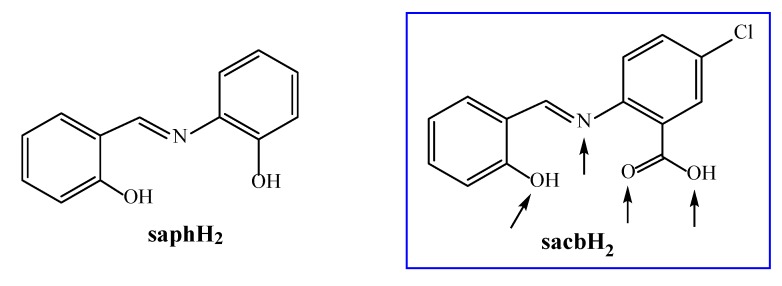
Structural formulae and abbreviations of the Schiff base ligands discussed in the text; the blue box encloses the tetradentate ligand sacbH_2_ used in the present work, whereas the arrows indicate the potential donor atoms.

Considering the significant effect of the organic chelate ligand on the synthesis of new coordination compounds with interesting structural and magnetic properties, our group has started a program aiming at the exploration of the coordination affinity of various Schiff base ligands, related to the parent *N*-salicylidene-*o*-aminophenol (saphH_2_, Scheme 1), with magnetically interesting 3d- and 4f-metal ions, and 3d/4f-metal combinations [19]. The ligand of choice for this work was *N*-salicylidene-2-amino-5-chlorobenzoic acid (sacbH_2_, Scheme 1), which—upon complete deprotonation—can act as a tetradentate (O_3_N) chelating and bridging ligand to both di- and trivalent metal ions, thus yielding structurally interesting polynuclear metal complexes. This has been clearly evidenced by the synthesis and characterization of several Ni^II^ and Ln^III^ complexes with unprecedented nuclearities (i.e., {Ni_18_} and {Ni_26_} clusters [20]) and exciting SMM properties (i.e., a large barrier of 109 K for a {Dy_2_} compound [21]). We herein report the first successful results from the initial employment of sacbH_2_ in the redox-active Mn coordination chemistry. In particular, we have used the self-assembly method to prepare and structurally characterize the mononuclear compound (Pr_2_NH_2_)[Mn^III^(sacb)_2_] (**1**) in high yields, and subsequently converted **1** to the 1-D polymer {[Mn^II^Mn^III^(sacb)_2_(H_2_O)_2_(MeOH)_2_](NO_3_)}_n_ (**2**) by adopting the ‘metal complexes as ligands’ strategy.

## 2. Materials and Methods

All manipulations were performed under aerobic conditions, using materials (reagent grade) and solvents as received unless otherwise noted. The Schiff base ligand sacbH_2_ was prepared, purified, and characterized as described elsewhere [20,21]. Infrared spectra were recorded in the solid state on a Bruker’s FT-IR spectrometer (ALPHA’s Platinum ATR single reflection) in the 4000–400 cm^−1^ range. Elemental analyses (C, H, and N) were performed on a Perkin-Elmer 2400 Series II Analyzer. Variable-temperature magnetic susceptibility studies were performed, using an MPMS5 Quantum Design magnetometer operating at 0.03 T in the 300–2.0 K range. Diamagnetic corrections were applied to the observed paramagnetic susceptibility, using Pascal’s constants [22].

### 2.1. Synthesis of (Pr_2_NH_2_)[Mn(sacb)_2_] (**1**)

To a stirred yellowish solution of sacbH_2_ (0.11 g, 0.40 mmol) and Pr_2_NH (82 μL, 0.60 mmol) in MeOH (10 mL) was added a pink solution of Mn(O_2_CMe)_2_∙4H_2_O (0.05 g, 0.20 mmol) in the same solvent (5 mL). The resulting orange–red solution was stirred for 12 h, during which time a red microcrystalline solid was formed. The reddish material was collected by filtration, washed with cold MeOH (2 × 3 mL), and dried under vacuum. Single crystals suitable for X-ray diffraction studies were grown from a slow evaporation process of the resulting filtrate, and the crystals were analyzed as **1**∙MeOH. The IR spectrum of the red microcrystalline material was identical to that of the vacuum-dried, single crystals of **1**. The yield of the red microcrystalline solid was 88% (based on the ligand available). The dried solid was satisfactorily analyzed as lattice-solvent free, i.e., as **1**. Anal. calc. for C_34_H_32_N_3_O_6_MnCl_2_ (found values in parentheses): C 57.97 (57.78), H 4.58 (4.45), N 5.96 (6.02) %. Selected IR data (ATR): *ν* = 3462 (mb), 2964 (w), 2777 (mb), 1635 (s), 1573 (s), 1551 (s), 1536 (m), 1435 (s), 1401 (m), 1350 (m), 1327 (m), 1287 (s), 1229 (w), 1186 (s), 1148 (m), 1122 (m), 1105 (m), 1030 (m), 988 (m), 967 (m), 923 (m), 883 (vs), 847 (w), 824 (m), 794 (m), 759 (vs), 731 (s), 664 (w), 636 (s), 617 (m), 600 (s), 563 (m), 547 (m), 537 (w), 497 (m), 445 (s), 428 (s).

### 2.2. Synthesis of {[Mn_2_(sacb)_2_(H_2_O)_2_(MeOH)_2_](NO_3_)}_n_ (**2**)

To a stirred, orange–red suspension of **1** (0.35 g, 0.50 mmol) in MeOH (10 mL) was added a pink solution of Mn(NO_3_)_2_∙4H_2_O (0.13 g, 0.50 mmol) in the same solvent (5 mL). The resulting dark-red suspension was stirred for 20 min, during which time all solids were dissolved, and the solution turned brownish and clear. The latter solution was filtered, and the filtrate was left to evaporate slowly at room temperature. After three days, X-ray-quality red plate-like crystals of **2**∙2H_2_O were formed, and these were collected by filtration, washed with cold MeOH (2 × 3 mL), and dried in air. The yield was 50% (based on the ligand available). The air-dried solid was satisfactorily analyzed as lattice-solvent free, i.e., as **2**. Anal. calc. for C_30_H_28_N_3_O_13_Mn_2_Cl_2_ (found values in parentheses): C 43.98 (43.77), H 3.44 (4.36), N 5.13 (5.22) %. Selected IR data (ATR): *ν* = 3392 (mb), 2921 (m), 2850 (m), 1604 (vs), 1585 (s), 1538 (s), 1485 (m), 1463 (m), 1437 (s), 1407 (m), 1384 (s), 1291 (m), 1248 (w), 1190 (m), 1152 (m), 1111 (m), 1030 (w), 994 (w), 981 (w), 928 (m), 894 (m), 854 (m), 827 (w), 797 (w), 759 (m), 731 (m), 718 (m), 641 (m), 606 (m), 567 (w), 550 (m), 451 (m).

### 2.3. Single-Crystal X-ray Crystallography

Crystals of the complexes **1**∙MeOH and **2**∙2H_2_O were selected and mounted on MiteGen dual thickness micromounts TM, using inert oil [23]. Diffraction data were collected on a D8 BRUKER diffractometer, equipped with a multilayer mirror monochromator and a Mo Kα microfocus sealed tube (λ = 0.71073 Å). Images were processed with the software SAINT+ [24], and absorption effects were corrected with the multi-scan method implemented in SADABS [25]. The structures were solved by using SHELXTL incorporated in the Bruker APEX-III software package and refined by using the SHELXLE and PLATON programs [26,27]. All the non-hydrogen atoms of the two structures were successfully refined by using anisotropic displacement parameters. Hydrogen atoms bonded to the carbon and nitrogen atoms of the ligands were placed at their idealized positions, using *HFIX* instructions in SHELXL, and included in subsequent refinement cycles in riding-motion approximation, with isotropic thermal displacements parameters (*U*_iso_) fixed at 1.2 or 1.5 × *U_eq_*. Furthermore, the H-atoms associated with the OH groups of water and methanol molecules were markedly visible in the difference Fourier maps, and they were included in subsequent refinement cycles, with the O–H and H···H distances restrained to 0.90(1) and 1.40(1) Å, respectively, and using a riding-motion approximation, with an isotropic thermal displacement parameter fixed at 1.5 × *U_eq_* of the parent oxygen atom. The program SQUEEZE, a part of the PLATON package of crystallographic software, was also used to remove the contribution from the additional disordered lattice solvate molecules. Various figures of the structures were created, using the Diamond 3 [28] and Mercury [29] software packages. Unit cell parameters, structure solution, and refinement details for **1**∙MeOH and **2**∙2H_2_O are summarized in Table 1. Further crystallographic details can be found in the corresponding CIF files provided in the ESI.

Crystallographic data (excluding structure factors) for both structures **1** and **2** were deposited with the Cambridge Crystallographic Data Centre (CCDC) as supplementary publication numbers: CCDC-1988621 (**1**) and CCDC-1980855 (**2**). Copies of these data can be obtained free of charge on application to CCDC, 12 Union Road, Cambridge CB2 2EZ, UK; FAX: (+44) 1223 336033, or online via www.ccdc.cam.ac.uk/data_request/cif or by emailing data_request@ccdc.cam.ac.uk.

## 3. Results and Discussion

### 3.1. Synthetic Comments

Most synthetic procedures to oligonuclear or polynuclear Mn compounds in moderate to high oxidation states (II, III, and IV, or mixed-valence) rely on the reactions of either the triangular [Mn_3_O(O_2_CR)_6_L_3_]^0,+^ precursors (RCO_2_^−^ = various carboxylates; L = terminal, neutral groups) or simple Mn^II^ salts with a potentially chelating/bridging ligand under aerobic conditions. In the absence of any previous use of the Schiff base ligand sacbH_2_ in Mn coordination chemistry, several reactions were explored, differing in the Mn^II^ starting materials, the nature of the external base, the Mn:sacbH_2_:base ratio, and/or the reaction solvent. Eventually, we were able to identify the following successful system, which provided us with the highest yields and purity of the resulting crystalline material. Therefore, the reaction between Mn(O_2_CMe)_2_∙4H_2_O, sacbH_2_, and Pr_2_NH, in a 1:2:3 molar ratio, in solvent MeOH, afforded red crystals of the mononuclear complex (Pr_2_NH_2_)[Mn(sacb)_2_] (**1**) in yields as high as 88%. The general formation of **1** is summarized by the following stoichiometric equation:

(1) $$\mathrm{Mn}2^{+}+2\;{\mathrm{sacbH}}_{2}+3\;{\mathrm{Pr}}_{2}\mathrm{NH}+\frac{1}{4}\;O_{2}\;\to \;[\mathrm{Mn}(\mathrm{sacb}{)}_{2}{]}^{-}+3\;{\mathrm{Pr}}_{2}{{\mathrm{NH}}_{2}}^{+}+\frac{1}{2}\;H_{2}O$$

The oxidation of Mn^II^ to Mn^III^ is almost certainly by atmospheric O_2_ under the prevailing basic conditions. Reactions in the absence of an external base gave only pale-yellow solutions indicative of exclusively Mn^II^-containing products, likely bearing the neutral sacbH_2_ ligand. The presence of Pr_2_NH is also essential for the clean isolation and high-yield synthesis of **1**, providing a proton acceptor to facilitate the complete deprotonation of sacbH_2_. Furthermore, the resulting Pr_2_NH_2_^+^ cations in solution were proved (vide infra) sterically and electronically ideal to counterbalance the anionic [Mn(sacb)_2_]^−^ species and foster the crystallization of **1**. Various similar reactions in other organic solvents (i.e., MeCN, EtOH, CH_2_Cl_2_, and DMF) and/or external bases (i.e., Et_3_N, Et_2_NH, Me_2_NH, and Me_3_N) yielded oily products that we were unable to further characterize and crystallize. Complex **1** can also be obtained with non-carboxylate Mn^II^ starting materials, such as MnCl_2_ and Mn(ClO_4_)_2_, but the yields were always much lower (<20%).

The high-yield synthesis of complex **1** and the fact that it contains coordinated sacb^2−^ ligands, but with dangling carboxylate O atoms (vide infra), probed us to use **1** as the building block for reactions with external Mn^II^ precursors. This synthetic approach of ‘metal complexes as ligands’ proved to be successful, and from the 1:1 reaction between (Pr_2_NH_2_)[Mn(sacb)_2_] (**1**) and Mn(NO_3_)_2_∙4H_2_O in solvent MeOH, we were able to isolate red crystals of the 1-D coordination polymer {[Mn_2_(sacb)_2_(H_2_O)_2_(MeOH)_2_](NO_3_)}_n_ (**2**) in ~50% yield. Complex **2** is mixed-valence Mn^II^Mn^III^, retaining the Mn oxidation states as appeared in the two individual reactants. The general formation of **1** is summarized by the following stoichiometric equation:

n [Mn(sacb)_2_]^−^ + n Mn^2+^ + 2n H_2_O + 2n MeOH → {[Mn_2_(sacb)_2_(H_2_O)_2_(MeOH)_2_]^+^}_n_ (2)

Once the identity of **2** had been established, we attempted to identify a more convenient preparation of **2** in MeOH, involving aerial oxidation of Mn(NO_3_)_2_∙4H_2_O in the presence of sacbH_2_ and different organic bases. Surprisingly, we were unable to isolate any crystalline material that could be identified as **2**, and our efforts always resulted in amorphous precipitates which were probably mixtures of different products. In our hands, it appears that the polymeric complex **2** can only be prepared in a pure and crystalline form by using complex **1** as the metalloligand and the Mn(NO_3_)_2_ as a dual provider of Mn^II^ ions and NO_3_^−^ counterions in solution (vide infra).

### 3.2. Description of Structures

**Figure 1:**
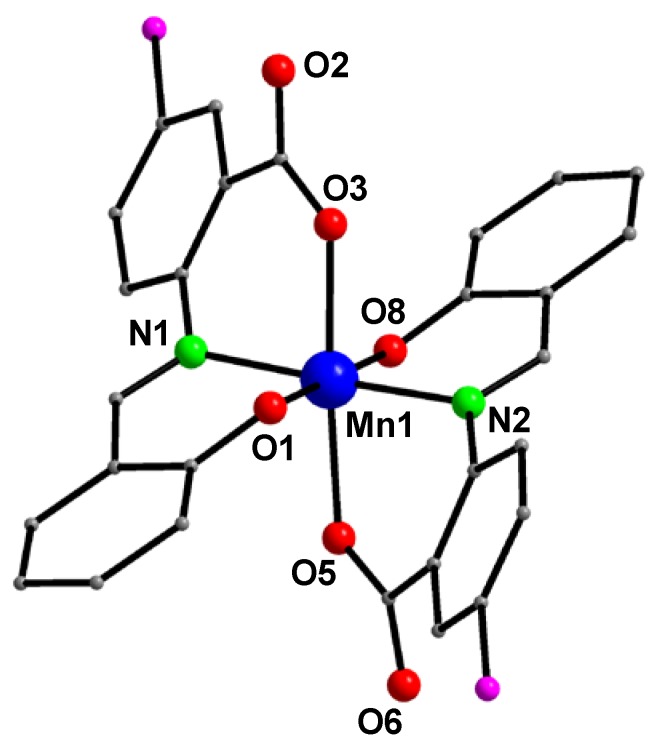
Partially labeled representation of the anion of complex **1**. Color scheme: Mn^III^, blue; Cl, purple; O, red; N, green; C, gray. H atoms are omitted for clarity.

A partially labeled representation of the anion of complex **1** is shown in Figure 1. The negatively charged [Mn(sacb)_2_]^−^ is counterbalanced by a Pr_2_NH_2_^+^ cation, and the overall compound crystallizes with a MeOH solvate molecule in the crystal lattice of **1**. Selected interatomic distances and angles of **1** are listed in Table 2.

The central Mn^III^ ion in **1** is surrounded by two O_c_N_im_O_p_-tridentate chelating sacb^2−^ ligands, where O_c_, N_im_, and O_p_ denote the coordinated carboxylate O, imine N, and phenoxido O donor atoms of sacb^2−^, respectively. The three donor atoms of the same sacb^2−^ ligand are in *facial*-position (O_c_−Mn−N_im_ = 82.2(8)/83.7(8)°, O_c_−Mn−O_p_ = 91.9(8)/91.6(8)°, and O_p_−Mn−N_im_ = 88.0(9)/87.7(9)°), while the chemically similar donor atoms of the two ligands are mutually *trans* to each other (O_c_−Mn−O_c_ = 175.8(7)°, N_im_−Mn−N_im_ = 177.3(9)°, and O_p_−Mn−O_p_ = 179.6(8)°). The salicylaldimine (O_p_C_7_N_im_) and iminocarboxylate (O_c_C_7_N_im_) fragments of each dianionic ligand form two very distorted six-membered chelate rings. The angles between the MnO_p_C_3_N_im_ and MnO_c_C_3_N_im_ chelate rings within each sacb^2−^ ligand are 57.9° and 57.1°. Thus, the Mn^III^ atom is six-coordinate with distorted octahedral geometry, and exhibits Jahn–Teller (JT) distortion, as expected for a d^4^ ion in near-octahedral geometry. The distortion takes the form of an axial elongation, as is almost always the case for Mn^III^, with the elongation axis being O_c_-Mn-O_c_, as reflected in the two long *trans* bonds (Mn1−O3 = 2.185(2) and Mn1−O5 = 2.154(2) Å). The Mn^III^ oxidation state was also confirmed by a bond valence sum (BVS) calculation [30], which gave a value of 3.06.

**Figure 2:**
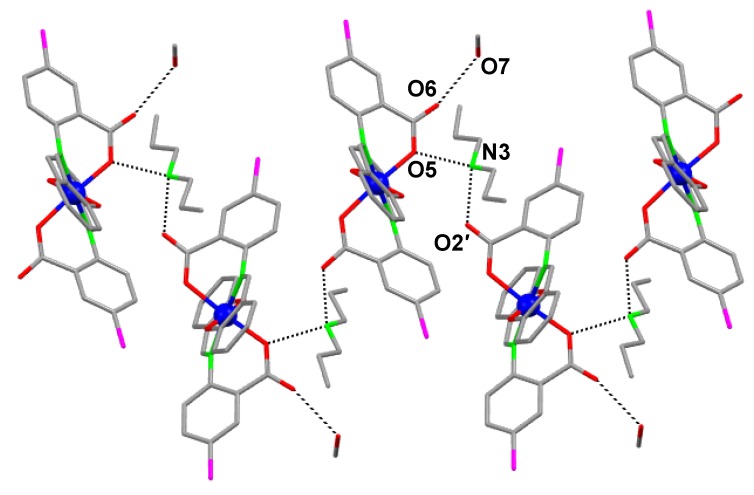
A small portion of the pseudo zig-zag chains formed through the H-bonding interactions (dashed lines) between the Pr_2_NH_2_^+^ counterions, the lattice MeOH molecules, and the carboxylate O atoms of sacb^2−^ in the crystal structure of **1**∙MeOH. H atoms are omitted for clarity. Color scheme as in Figure 1.

In the crystal structure of **1**∙MeOH, there are some intra- and intermolecular H-bonding interactions which deserve further discussion. In particular, the Pr_2_NH_2_^+^ counterion is doubly H-bonded to the coordinated carboxylate O atom of a sacb^2−^ ligand (N3−H26∙∙∙O5 = 2.821(2) Å) and the dangling carboxylate O atom of a different sacb^2−^ ligand (N3−H25∙∙∙O2′ = 2.842(3) Å) from a neighboring molecule. This illustrates the structural importance of the Pr_2_NH_2_^+^ counterion in not only balancing the negative charge of the [Mn(sacb)_2_]^−^ complex anion but also holding the molecules together in the crystal through an extensive array of H-bonding interactions (Figure 2). In addition, the lattice MeOH solvate molecule is H-bonded to the dangling carboxylate O atom of the remaining sacb^2−^ ligand (O7-H77∙∙∙O6 = 2.740(3) Å). No other significant H-bonding or π···π stacking interactions were observed in the crystal structure of **1**∙MeOH. It should be noted that Alborés and co-workers have recently reported a structurally unique [Mn^III^L(LH)] monomer, where LH_2_ is a Schiff base ligand derived from the condensation of salicylaldehyde and anthranilic acid, which is organized into 1-D hydrogen-bonded chains [31].

**Figure 3:**
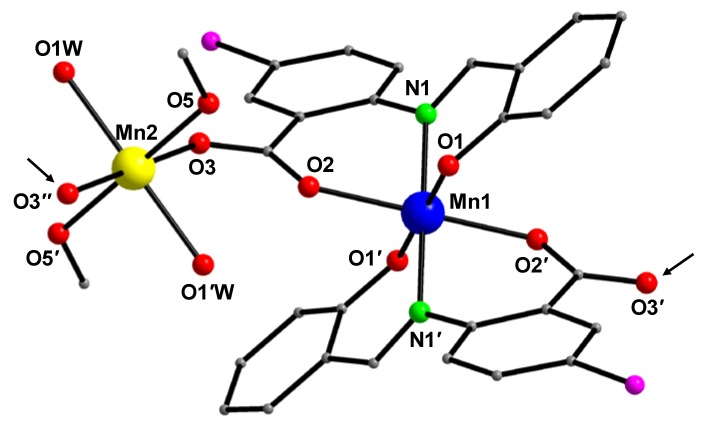
Partially labeled representation of the [Mn_2_(sacb)_2_(H_2_O)_2_(MeOH)_2_]^+^ repeating unit of the 1-D polymer **2**. Color scheme: Mn^III^, blue; Mn^II^, yellow; Cl, purple; O, red; N, green; C, gray. H atoms are omitted for clarity. Primed and unprimed atoms are related by symmetry. The arrows indicate the O atoms, which are responsible for the polymerization of the dinuclear repeating units.

The removal of the Pr_2_NH_2_^+^ counterion from the vicinity of the [Mn(sacb)_2_]^−^ compound resulted in the coordination of the dangling carboxylate O atom of sacb^2−^ to adjacent {Mn(MeOH)_2_(H_2_O)_2_}^2+^ units, and subsequently the formation of the 1-D coordination polymer {[Mn^II^Mn^III^(sacb)_2_(H_2_O)_2_(MeOH)_2_](NO_3_)}_n_ (**2**). The crystal structure of **2**∙2H_2_O consists of the ions [Mn_2_(sacb)_2_(H_2_O)_2_(MeOH)_2_]^+^ and NO_3_^−^, and solvate H_2_O molecules in an 1:1:1 ratio. The [Mn_2_(sacb)_2_(H_2_O)_2_(MeOH)_2_]^+^ repeating unit of the 1-D polymer **2** is shown in Figure 3. Selected interatomic distances and angles of **2** are listed in Table 3.

The removal of the Pr_2_NH_2_^+^ counterion from the vicinity of the [Mn(sacb)_2_]^−^ compound resulted in the coordination of the dangling carboxylate O atom of sacb^2−^ to adjacent {Mn(MeOH)_2_(H_2_O)_2_}^2+^ units, and subsequently the formation of the 1-D coordination polymer {[Mn^II^Mn^III^(sacb)_2_(H_2_O)_2_(MeOH)_2_](NO_3_)}_n_ (**2**). The crystal structure of **2**∙2H_2_O consists of the ions [Mn_2_(sacb)_2_(H_2_O)_2_(MeOH)_2_]^+^ and NO_3_^−^, and solvate H_2_O molecules in an 1:1:1 ratio. The [Mn_2_(sacb)_2_(H_2_O)_2_(MeOH)_2_]^+^ repeating unit of the 1-D polymer **2** is shown in Figure 3. Selected interatomic distances and angles of **2** are listed in Table 3.

**Figure 4:**
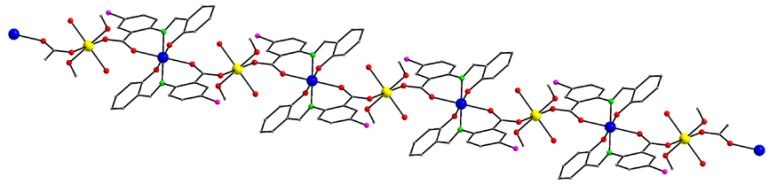
Representation of a section of the 1-D chain of **2**, showing the alternating {Mn^III^(sacb)_2_}^−^ and {Mn^II^(MeOH)_2_(H_2_O)_2_}^2+^ subunits. Color scheme as in Figure 3.

The [Mn^III^(sacb)_2_]^−^ building block that appears in the repeating unit of **2** is structurally very similar to the discrete molecular unit of **1**; the *facial* arrangement of the donor atoms that form the chelate rings, the Mn^III^ oxidation state (BVS of Mn1 = 3.15), and the JT elongation axis (Mn1-O_c_ = 2.144(1) Å and O_c_-Mn1-O_c_ = 180°) have been nearly unaltered. However, the second carboxylate O atom of each sacb^2−^ in the [Mn^III^(sacb)_2_]^−^ unit is now bound to a Mn^II^ ion (Mn2, BVS = 1.98) in an *anti*-fashion, and this linkage is infinitely repeated to yield an 1-D, linear chain (Mn^II^∙∙∙Mn^III^∙∙∙Mn^II^ and Mn^III^∙∙∙Mn^II^∙∙∙Mn^III^ angles = 180°) that runs parallel to the *c*-axis (Figure 4). Thus, the two sacb^2−^ ligands in **2** act as tetradentate chelating and bridging groups, binding in an overall η^1^:η^1^:η^1^:η^1^:μ mode. The remaining coordination sites of the almost ideal octahedral Mn^II^ ion are occupied by the O atoms of two terminally bound MeOH (O5/O5′) and H_2_O (O1W/O1′W) molecules. The *trans* O-Mn1-O and N-Mn1-N angles are all 180°, while the *cis* O-Mn1-O and N-Mn1-O angles span the range 88.2–96.5°. The Mn1∙∙∙Mn2 distance within the 1-D-chain of **2** is 5.488(4) Å, thus presaging very weak magnetic exchange interactions between the paramagnetic metal ions. Furthermore, the shortest Mn∙∙∙Mn separation between the chains is 8.3 Å (Supplementary Figure S1), and no significant inter-chain H-bonding or π-π stacking interactions appear to affect the structural isolation of the 1-D polymers, other than some weak contacts between the NO_3_^−^ counterions and the lattice H_2_O solvate molecules. The NO_3_^−^ ions and the lattice H_2_O molecules appear to occupy the voids between adjacent 1-D chains. Interestingly, there are some strong intra-chain H-bonding interactions that involve the coordinated O atoms of sacb^2−^, MeOH and H_2_O molecules, and the lattice NO_3_^−^ and H_2_O groups; these interactions serve to stabilize the {Mn(MeOH)_2_(H_2_O)_2_}^2+^ units within the polymer of **2** (Supplementary Figure S2).

Finally, it is worth mentioning that, although there are thousands of Mn(II) polymers reported in the literature to date, the mixed-valence Mn(II/III) coordination polymers are still very rare [32,33]. This is likely due to the preference of Mn^III^ ions to form 0-D coordination clusters with bridging O^2−^ and OR^−^ groups (HSAB principle), in the presence of several different chelates. The vast majority of the previously reported Mn(II/III) polymers includes products that have been resulted from the polymerization of high-nuclearity {Mn_x_} cluster compounds (x = 3, 6, 12, etc.) [34,35,36,37]. The structurally closest to our reported compound **2** is the 1-D linear chain of [Mn^III^(4-Me-sal)_2_(4-Me-py)_2_Mn^II^(H_2_O)_2_(MeOH)_2_]+ repeating units [38], which includes the tridentate chelating/bridging ligand 4-methyl salicylic acid (4-Me-salH_2_) and terminally bound 4-methyl pyridine (4-Me-py), H_2_O, and MeOH molecules. The cationic 1-D chain is mixed-valence, with alternating Mn^III^ and Mn^II^ ions, and the bridging between the metal ions is exclusively provided by a *syn,anti*-carboxylate group. The magnetic study of this 1-D chain revealed the presence of weak antiferromagnetic exchange interactions between the Mn ions and a small *J*-coupling constant of −0.55 K.

### 3.3. IR Spectroscopy

**Figure 5:**
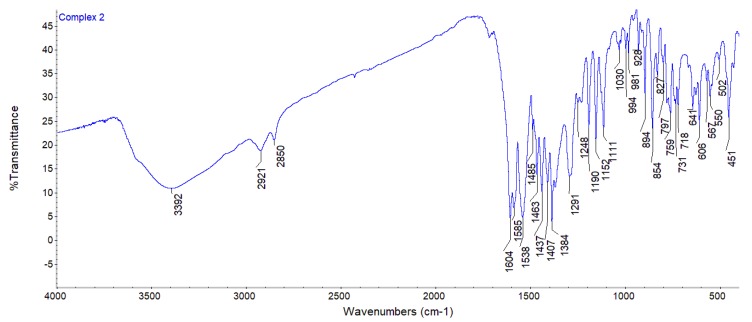
FT-IR spectrum of complex **2**∙2H_2_O.

Since complex **2** is chemically and structurally the most salient feature of this work, and provided that part of complex **1** exists as a building block within the 1-D polymer **2**, we restrict the IR spectroscopic discussion to the latter compound. The IR spectrum of freshly prepared **2**∙2H_2_O (Figure 5) exhibits medium intensity bands in the 3400–2850 cm^−1^ region assignable to *ν*(OH) from MeOH and H_2_O molecules (both coordinated and lattice). The broadness and low frequency of these bands are both indicative of hydrogen bonding [39]. The presence of ionic NO_3_^−^ in the structure of **2** is accompanied by the clear appearance of the *ν*_3_(E′)[*ν*_d_(NO)] IR-mode of the *D*_3h_ ionic nitrate at ~1384 cm^−1^ [40]. The very strong band at 1604 cm^−1^ is assigned to the C=N stretching vibration of the Schiff base linkage, *ν*(C=N) [41]. This band has been shifted to lower frequencies on going from the free ligand sacbH_2_ [*ν*(C=N) = 1620 cm^−1^] to the complex **2**; this is due to the coordination of the imino N atom to Mn^III^ [41]. The band at 1291 cm^−1^ is associated with phenolate-type C-O stretching vibrations [42].

### 3.4. Solid-State Magnetic Susceptibility Studies

**Figure 6:**
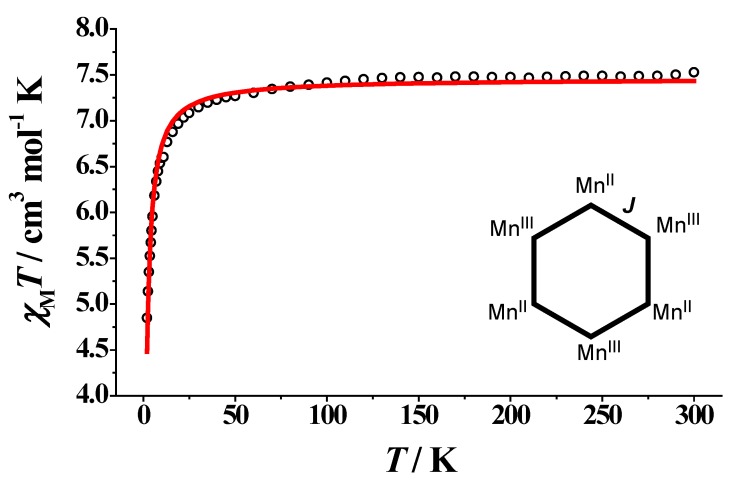
*χ*_M_*T* versus *T* plot for complex **2**. The solid red line is the fit of the data; see the text for the spin Hamiltonian and the corresponding fit parameters. (inset) *J*-coupling scheme employed for the determination of the fit parameters.

Direct-current (dc) magnetic susceptibility measurements on a crystalline sample of the potentially interesting (from a magnetism viewpoint) complex **2** were performed in the 2–300 K range, under an applied magnetic field of 0.03 T (Figure 6). The room-temperature *χ*_Μ_*T* value of 7.52 cm^3^Kmol^−1^ is very close to the theoretical value of 7.375 cm^3^Kmol^−1^ for two non-interacting Mn^II^ and Mn^III^ ions (*g* = 2.00) of the 1-D repeating unit. The *χ*_Μ_*T* product remains almost constant in the 300–100 K range, and upon further cooling, it steadily decreases down to a value of 4.85 cm^3^Kmol^−1^ at 2 K. The overall magnetic response of **2** is suggestive of the presence of weak antiferromagnetic exchange interactions between the metal ions, as expected for a system where the alternating Mn^II^ and Mn^III^ ions are solely bridged by a *syn-anti* carboxylate group [38,43].

To evaluate the intrachain exchange coupling constant in **2**, the system was treated as a simplified six-spin model, comprising a closed ring of alternating Mn^II^∙∙∙Mn^III^ paramagnetic ions (inset of Figure 6). Similar magnetic models have been used for the simulation of the susceptibility data of various 1-D chains [44,45]. The *χ*_Μ_*T* versus *T* data were fitted (red line in Figure 6) to the spin Hamiltonian given by Equation (3), where *J* coupling constant represents the interaction between adjacent *S*_1,3,5_ = 5/2 (for Mn^II^) and *S*_2,4,6_ = 2 (for Mn^III^) spin carriers.

H = −2*J*(*Ŝ*_1_·*Ŝ*_2_ + *Ŝ*_2_·*Ŝ*_3_ + *Ŝ*_3_·*Ŝ*_4_ + *Ŝ*_4_·*Ŝ*_5_ + *Ŝ*_5_·*Ŝ*_6_ + *Ŝ*_6_·*Ŝ*_1_) (3)

The best-fit parameters were *J* = −0.08 cm^−1^ and *g* = 2.00, in agreement with the expected, weak intrachain interactions between the metal ions. The very long separation (>10 Å) between next-neighboring Mn^II^∙∙∙Mn^II^ and Mn^III^∙∙∙Mn^III^ intra- and inter-chain pairs renders ineffective the presence of any additional magnetic exchange interaction. Given the simplicity of the employed magnetic model and the resulting good fit of the corresponding data, the magnetic anisotropy of the Mn^III^ metal ions has been neglected and is included phenomenologically in the coupling parameter, *J*, that is therefore probably slightly overestimated [38]. Finally, the experimental data have been also fitted to a Curie–Weiss law in the temperature region 2–300 K, leading to a Curie constant (*C*) of 7.53 cm^3^Kmol^−1^ and a Weiss constant (*θ*) of −1.4 K (Supplementary Figure S3). The Curie constant is in agreement with the expected value of 7.375 cm^3^Kmol^−1^ for one Mn^II^ and one Mn^III^ ions at high temperatures. On the other hand, the negative and small-in-magnitude *θ* further confirms the presence of very weak and antiferromagnetic interactions between the spin carriers.

## 4. Conclusions

In conclusion, we have shown in this work that the ‘metal complexes as ligands’ approach is a fruitful synthetic strategy for the isolation of new Mn^III^-containing coordination polymers with interesting structural and physicochemical properties. By using the [Mn^III^(sacb)_2_]^−^ metalloligand, which was made serendipitously via the aerial oxidation of a Mn^II^ precursor in the presence of the Schiff base ligand sacbH_2_, we managed to unveil the coordination of the dangling carboxylate O atoms of sacb^2−^ to adjacent {Mn(MeOH)_2_(H_2_O)_2_}^2+^ units, and subsequently form the 1-D polymer {[Mn^III^(sacb)_2_Mn^II^(MeOH)_2_(H_2_O)_2_](NO_3_)}_n_ in good yields. The 1-D polymer **2** has a linear chain conformation and consists of alternating Mn^II^ and Mn^III^ paramagnetic metal ions, which appear to be weakly and antiferromagnetically coupled through the *syn*-*anti* carboxylate groups of sacb^2−^ ligands. These results demonstrate, for the first time, the ability of sacbH_2_ to stabilize redox-active 3d-metal ions in moderate to high oxidation states, thus complementing our previous successful results from the employment of this ligand in Ni^II^ and Dy^III^ coordination chemistry [20,21,46].

We are currently seeking ways of deliberately replacing the Mn^II^ ions in **2** by other divalent 3d-metal ions with appreciable magnetic anisotropy, such as Co^II^, in an attempt to prepare new families of SCMs. Furthermore, we are expanding the coordination chemistry of sacbH_2_ to heterometallic 3d/4f-metal complexes as a means of obtaining molecular materials with new nanosized structures and exciting magnetic, optical, and catalytic properties. Both of these directions are in progress, and the results will be reported in due course.

## Figures and Tables

**Table 1:** Crystallographic data for complexes **1**∙MeOH and **2**∙2H_2_O.

Parameter	1∙MeOH	2∙2H_2_O
Empirical formula	C_35_H_36_N_3_O_7_MnCl_2_	C_30_H_32_N_3_O_15_Mn_2_Cl_2_
FW/g mol^−1^	736.51	855.36
Temperature/K	150(2)	150(2)
Crystal system	Monoclinic	Triclinic
Space group	*P*2_1_/*c*	*P*-1
*a*/Å	14.594(5)	8.298(7)
*b*/Å	12.770(5)	10.975(7)
*c*/Å	19.715(7)	12.548(13)
*α*/°	90	69.85(3)
*β*/°	109.561(9)	79.84(4)
*γ*/°	90	87.23(2)
Volume/Å^3^	3462(2)	1055.9(16)
*Z*	4	1
*ρ*_calc_/g cm^−3^	1.413	1.345
*μ*/mm^−1^	0.588	0.787
F(000)	1528	437
Radiation	Mo Kα (*λ* = 0.71073)	Mo Kα (*λ* = 0.71073)
Index ranges	−18 ≤ *h* ≤ 18	−9 ≤ *h* ≤ 9
	−15 ≤ *k* ≤ 15	−13 ≤ *k* ≤ 13
	−24 ≤ *l* ≤ 24	−14 ≤ *l* ≤ 14
Reflections collected	107100	29694
Data/restraints/parameters	7021/0/437	3705/7/277
Goodness-of-fit on *F*^2^	1.145	1.068
Final R indexes [*I* ≥ 2σ(*I*)] *^a,b^*	*R*_1_ = 0.0416	*R*_1_ = 0.0455
	w*R*_2_ = 0.1035	w*R*_2_ = 0.1199
Final R indexes [all data]	*R*_1_ = 0.0756	*R*_1_ = 0.0564
	w*R*_2_ = 0.1297	w*R*_2_ = 0.1257
(Δ*ρ*)_max,min_/e Å^−3^	0.828 and −0.592	1.082 and −0.312

*^a^ R*_1_ = ∑(||*F_o_*| – |*F_c_*||)/∑|*F_o_*|. *^b^* w*R*_2_ = [∑[*w*(*F*_o_^2^ - *F*_c_^2^)^2^]/∑[*w*(*F*_o_^2^)^2^]^1/2^, *w* = 1/[*σ* ^2^(*F*_o_^2^) + (*ap*)^2^ + *bp*], where *p* = [max(*F*_o_^2^, 0) + 2*F*_c_^2^]/3.

**Table 2:** Selected interatomic distances (Å) and angles (°) for complex **1**∙MeOH.

Mn1-O1	1.883(2)	Mn1-O8	1.882(2)
Mn1-O3	2.185(2)	Mn1-N1	2.054(2)
Mn1-O5	2.154(2)	Mn1-N2	2.049(2)
O8-Mn1-O1	179.6(8)	N2-Mn1-O5	83.7(8)
O8-Mn1-N2	87.7(9)	N1-Mn1-O5	93.7(8)
O1-Mn1-N2	92.6(9)	O8-Mn1-O3	88.0(8)
O8-Mn1-N1	91.5(9)	O1-Mn1-O3	91.9(8)
O1-Mn1-N1	88.0(9)	N2-Mn1-O3	100.3(8)
N2-Mn1-N1	177.3(9)	N1-Mn1-O3	82.2(8)
O8-Mn1-O5	91.6(8)	O5-Mn1-O3	175.8(7)
O1-Mn1-O5	88.4(8)		

**Table 3:** Selected interatomic distances (Å) and angles (°) for the 1-D polymer **2**∙H_2_O *^a^*.

Mn1-O1	1.894(3)	Mn2-O1W	2.180(3)
Mn1-O2	2.144(2)	Mn2-O3	2.160(3)
Mn1-N1	2.023(3)	Mn2-O5	2.189(3)
O1-Mn1-O2	92.1(1)	O1W-Mn2-O3	91.9(1)
O1-Mn1-O2′	107.9(2)	O1W-Mn2-O3′′	88.1(1)
O1-Mn1-N1	109.9(2)	O1W-Mn2-O5	90.3(1)
O1-Mn1-N1′	108.6(2)	O1W-Mn2-O5′	89.7(1)
O2-Mn1-N1	103.0(2)	O3-Mn2-O5	89.9(1)
O2-Mn1-O1′	97.0(2)	O3-Mn2-O5′	90.1(1)

*^a^* Symmetry codes: ′ = -*x*, -*y*, -*z*; ′′ = -*x*, -*y*, 1-*z*.

## Supplementary Materials

The following are available online at https://www.mdpi.com/1996-1944/13/6/1352/s1. Figure S1: Parallel arrangement of adjacent 1-D linear chains of **2** along the *c*-axis. Color scheme: Mn^III^, blue; Mn^II^, yellow; Cl, purple; O, red; N, green; C, gray. H atoms are omitted for clarity. Figure S2: A small section of the 1-D chain of **2**∙2H_2_O, emphasizing with dashed lines the intra-chain H-bonding interactions, which are discussed in the text. Color scheme as in Figure S1; H atoms are shown in cyan. Figure S3: Plot of 1/*χ*_M_ versus *T* for complex **2**. The solid red line is the fit of the data to the Curie–Weiss law; see the text for the corresponding fit parameters.
