# Supplementary material for: ‘Metal Complexes as Ligands’ for the Synthesis of Coordination Polymers: A MnIII Monomer as a Building Block for the Preparation of an Unprecedented 1-D {MnIIMnIII}n Linear Chain
Source: Materials (Basel). 2020 Mar 17;13(6):1352. doi: 10.3390/ma13061352 (PMC7143807; doi:10.3390/ma13061352)
Supplement: Supplementary file 1 [file materials-13-01352-s001.pdf]

materials

Supplementary Materials: 'Metal Complexes as Ligands' for the Synthesis of Coordination Polymers: A Mn^{III} Monomer as a Building Block for the Preparation of an Unprecedented 1-D $\{Mn^{II}Mn^{III}\}_n$ Linear Chain

Konstantinos N. Pantelis, Georgios Karotsis, Christos Lampropoulos, Luís Cunha-Silva, Albert Escuer and Theocharis C. Stamatatos

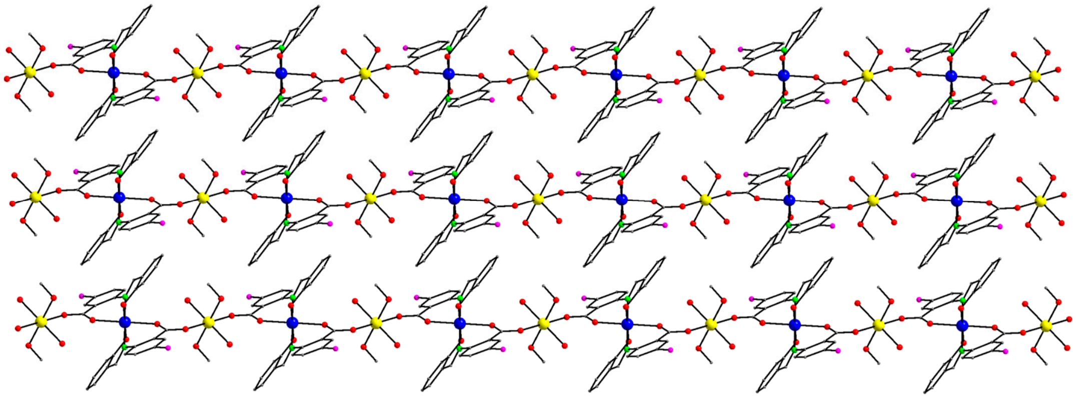

Figure S1. Parallel arrangement of adjacent 1-D linear chains of **2** along the *c*-axis. Color scheme: Mn^{III}, blue; Mn^{II}, yellow; Cl, purple; O, red; N, green; C, gray. H atoms are omitted for clarity.

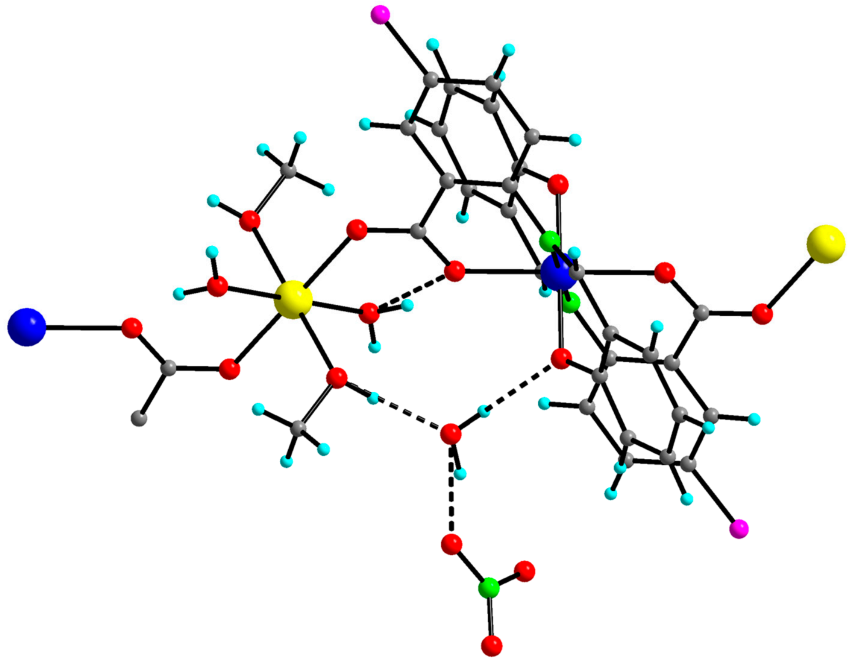

Figure S2. A small section of the 1-D chain of **2**·$2H_2O$, emphasizing with dashed lines the intra-chain H-bonding interactions which are discussed in the text. Color scheme as in Figure S1; H atoms are shown in cyan.

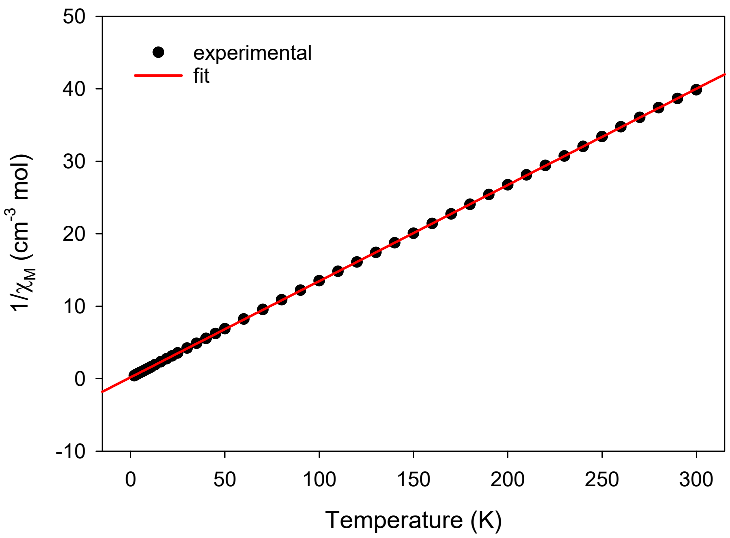

Figure S3. Plot of $1/\chi_M$ versus T for complex **2**. The solid red line is the fit of the data to the Curie-Weiss law; see the text for the corresponding fit parameters.

© 2020 by the authors. Submitted for possible open access publication under the terms and conditions of the Creative Commons Attribution (CC BY) license (http://creativecommons.org/licenses/by/4.0/).
